# Comparison of Hearing Outcomes in Tympanoplasty With and Without Ossiculoplasty: A Retrospective Cohort Study

**DOI:** 10.7759/cureus.73924

**Published:** 2024-11-18

**Authors:** Abdulrahman Alosaimi, Fatima N Abulfateh, Fahad K Bedawi, Aysha M Aljeeb, Omar A Sabra

**Affiliations:** 1 Otolaryngology - Head and Neck Surgery, Ohud Hospital, Medina, SAU; 2 Otolaryngology, Bahrain Defence Force Hospital, Manama, BHR; 3 Otolaryngology - Head and Neck Surgery, King Hamad University Hospital, Manama, BHR; 4 Medicine, Arabian Gulf University, Manama, BHR

**Keywords:** air-bone gap, cholesteatoma, middle ear risk index, ossiculoplasty, tympanoplasty

## Abstract

Objectives

To examine the ability of the Middle Ear Risk Index (MERI) score components in order to predict postoperative air-bone gap (ABG) and success rate in patients who underwent tympanoplasty.

Methods

A retrospective cohort study was conducted at King Hamad University Hospital between May 2017 and February 2021. A total of 79 patients were divided into two groups: 42 patients (53.2%) underwent tympanoplasty without ossiculoplasty, and 37 patients (46.8%) underwent tympanoplasty with ossiculoplasty. Data collected included demographic information, four-frequency ABG pre- and post-surgery, and total MERI scores. Statistical analyses included paired sample t-tests to assess changes in ABG and chi-square tests to evaluate associations between categorical variables. A p-value of <0.05 was considered significant.

Results

Seventy-nine patients were evaluated. Over half (53.2%) underwent myringoplasty only, and 46.8% (n = 37) underwent tympanoplasty with ossiculoplasty. Post-operative ABG was highly correlated with the presence of cholesteatoma, ossicular chain score, and the presence of previous surgery. The MERI score was correlated with the postoperative ABG gap, but with a relatively poor correlation coefficient, making it nonpractical for clinical use. Ossicular chain score alone was a better indicator of postoperative ABG. Successful closure of ABG was high in both groups, reaching 83.6% (n = 31) in the ossiculoplasty group and 92.8% (n = 39) in the myringoplasty group. Some components of the MERI score were found to be significantly interrelated, making the MERI score not a reliable prognostic indicator. Patients who underwent tympanoplasty with ossiculoplasty had high pre- and postoperative ABG values (27.47 ± 14.22 and 15.02 ± 8.58, respectively). The mean total MERI score (5.56 ± 2.24) was also significantly higher in patients who underwent ossiculoplasty.

Conclusions

Our findings indicate that both cholesteatoma and ossicular pathology significantly influence hearing outcomes, with a high degree of interrelation. While the MERI provides some insight, its limited predictive value, due to the interdependence of its components, suggests that it may not be a reliable tool for clinical decision-making in tympanoplasty and ossiculoplasty.

## Introduction

Tympanoplasty is a common surgery in otolaryngology [1], mainly used for repairing the tympanic membrane (TM) [2] and the restoration of middle ear function [3]. It is associated with chronic suppurative otitis media [4], which is commonly seen with dysfunction of the Eustachian tube [5]. Ossiculoplasty is necessary for the restoration of effective sound transmission when erosion or calcification of the ossicular chain, comprising the malleus, incus, and stapes, occurs as a result of chronic infections or cholesteatoma [6]. In addition, combining ossiculoplasty with tympanoplasty for the reconstruction of the ossicular chain optimizes the outcome of hearing [7]. Traumatic ossicular damage, although rare, further complicates middle ear repair [8].

The outcome of tympanoplasty with or without ossiculoplasty still remains inconsistent [1] and varies in outcomes [9]. Previous studies showed that myringoplasty alone, which is a condition where the ossicular chains remain intact, often results in very good postoperative auditory thresholds [10]. The outcome is predictable; however, it sometimes damages the ossicles, which warrants the undertaking of ossiculoplasty [11]. Variability in surgical technique, prosthetic materials, and residual eustachian tube dysfunction may account for the outcome variability. Complications from surgery, such as poor middle ear ventilation, scarring, and mucosal hyperplasia, may also further limit hearing gains [12]. This understanding highlights myringoplasty as an appropriate reference group when assessing ossiculoplasty hearing outcomes, given that both groups typically experience similar chronic Eustachian tube dysfunction, which may continue to impact hearing results post-surgery. Several scoring systems have been developed to assess the severity of middle ear disease and predict surgical outcomes, including the Ossiculoplasty Outcome Parameter Staging (OOPS), the Middle Ear Risk Index (MERI), and the Surgical, Prosthetic, Infection, Tissue, and Eustachian (SPITE) scores [13]. All of these scores include factors describing the status of sound-transmitting organs (drum and ossicular chain) and factors describing the status of the middle ear medium (otorrhea, fibrosis, granulation, etc.) [14]. These factors, along with other components of the scoring systems, predict the success of surgery, audiologically [15] and anatomically [16]. The MERI is crucial in predicting surgical outcomes [17], with critical risk factors that assess the status of cholesteatoma, middle ear mucous membrane, and ossicular chain [18]. The score generated with MERI has been used extensively to guide preoperative decision-making and predict surgical success for middle ear surgeries [19]. The ossicular chain status is one component of the MERI score but at the same time is the reason for ossiculoplasty. This component is believed to be related to other components of the scoring system, as most of these components are the result of eustachian tube dysfunction. This interrelation makes score components confounding factors, and as a result, adding up the scores of different components within the MERI score potentially exaggerates the severity of middle ear disease.

While several studies have explored the outcomes of tympanoplasty with or without ossiculoplasty, most are flawed by uncontrolled confounding factors, such as the effect of the ossicular chain itself on hearing performance [20]. These limits the verification of the potential of ossiculoplasty in patients with different risk profiles of their middle ear [21]. For example, Kotzias et al. reported better hearing results for the patients who undergo ossiculoplasty but could not fully demonstrate the effect of other conditions of the middle ear, which would limit the interpretation of the findings [22]. However, preoperative risk factors, such as the variation in MERI scores, were not controlled.

Further research is still needed to determine the impact of specific middle ear conditions on surgical outcomes to help refine these prognostic tools.

The first aim of this article is to describe the audiological air-bone gap (ABG) results in terms of success closure to within 20 dB and the mean ABG after ossiculoplasty and compare those results to results of myringoplasty as a control population. The second aim is to describe the relation between components of the preoperative MERI scores and postoperative ABG in both groups and the whole tympanoplasty population. The third aim is to study the possible interrelation between different components of the MERI score and how this would affect the ultimate predicting power of the MERI score in terms of hearing results.

## Materials and methods

Study design and settings

A retrospective cohort study was conducted at King Hamad University Hospital (KHUH) between May 2017 and February 2021.

Population

The study included adult patients (aged ≥18 years) of both genders who attended the ENT clinic at KHUH and underwent tympanoplasty with or without ossiculoplasty using a titanium total or partial ossicular replacement prosthesis (TORP or PORP). Patients with stapes fixation requiring stapedectomy or malleovestibulopexy were excluded due to the specific nature of these procedures and the potential for improved hearing outcomes compared to PORP and TORP reconstruction.

Sampling

A systematic sampling technique was employed to select participants. Specifically, every third patient meeting the inclusion criteria during the study period was chosen. This method ensured an unbiased and representative sample of the population undergoing tympanoplasty procedures at KHUH.

Surgical procedures

The sample comprised 79 patients divided into two groups: 42 patients (53.2%) underwent tympanoplasty without ossiculoplasty, and 37 (46.8%) underwent tympanoplasty with ossiculoplasty. The decision to perform ossiculoplasty was made intraoperatively based on a real-time assessment of ossicular integrity and mobility.

Data collection

Data were extracted from patients’ medical records. They included demographic information (age, gender, and smoking status), clinical findings (TM perforation, otorrhea, presence of cholesteatoma, middle ear mucosa status, and history of previous ear surgeries), four-frequency ABG measurements pre- and post-surgery, and total MERI scores. For this study, the MERI score was modified by excluding the ossicular chain component to isolate the impact of other middle ear conditions on hearing outcomes. Cases with incomplete records were excluded from the analysis. Five patients with missing ossicular chain scores were excluded from related analyses to maintain data integrity. No data imputation was performed.

Data analysis

Data were entered into Microsoft Excel (Microsoft Corporation, Redmond, WA, USA) and analyzed using IBM SPSS Statistics for Windows, Version 22.0 (Released 2013; IBM Corp., Armonk, NY, USA). Descriptive statistics were used to summarize demographic and clinical characteristics. Paired sample t-tests were utilized to assess changes in ABG values pre- and post-surgery within each group, providing insights into the effectiveness of the surgical interventions. Chi-square tests were employed to evaluate associations between categorical variables, such as the presence of cholesteatoma and the type of procedure performed. These statistical tests were chosen based on their appropriateness for comparing means and assessing categorical associations in the study design. A p-value of <0.05 was considered statistically significant. No adjustments for multiple comparisons were made, as the primary outcomes were limited to ABG changes and associations with critical clinical factors.

Protocol and registration

The study protocol was reviewed and approved by the Institutional Review Board (IRB) of KHUH (reference no. 22-528). All collected data were anonymized to maintain patient confidentiality.

## Results

General demographics

The study included 79 patients with a mean age of 42.34 ± 16.67 years. Among these patients, 56 (70.9%) had TM perforation. Otorrhea was present in 30 patients (38.0%), and cholesteatoma was diagnosed in 21 patients (26.6%). The middle ear mucosa was normal in 65 patients (82.3%), while 13 patients (16.5%) exhibited granulation or effusion. The included patients’ detailed demographic and clinical characteristics are presented in Table 1.

**Table 1:** Demographic and clinical characteristics of included patients ABG, air-bone gap; MERI, Middle Ear Risk Index; TM, tympanic membrane

Variable	Mean ± SD (%)/n
Age	42.34 ± 16.67
TM perforation	
Yes	56 (70.9%)
No	23 (29.1%)
Otorrhea	
Absent	49 (62.0%)
Present	30 (38.0%)
Cholesteatoma	
Yes	21 (26.6%)
No	58 (73.4%)
Ossicular chain score	
0	40 (50.6%)
1	6 (7.6%)
2	21 (26.6%)
3	11 (13.9%)
4	None
Missing cases (5)	40 (50.6%)
Middle ear mucosa	
Normal	65 (82.3%)
Granulation/effusion	13 (16.5%)
Tympanoplasty without ossiculoplasty	37 (46.8%)
Tympanoplasty with ossiculoplasty	42 (53.2%)
Missing case (1)	0
Previous surgery	
Yes	26 (32.9%)
No	53 (67.1%)
Smoker	
Yes	9 (11.4%)
No	70 (88.6%)
Total MERI score	3.57 ± 2.62
Modified MERI score	2.20 ± 1.54
Pre-op ABG	23.68 ± 12.33
Post-op ABG	12.10 ± 7.92

Of the total sample, 42 patients (53.2%) underwent tympanoplasty without ossiculoplasty, while 37 patients (46.8%) underwent tympanoplasty with ossiculoplasty. The mean MERI score was 3.57 ± 2.62 for the tympanoplasty-only group and 2.20 ± 1.54 for the ossiculoplasty group. The mean preoperative ABG was 23.68 ± 12.33 dB, and the mean postoperative ABG was 12.10 ± 7.92 dB (Table 1).

Demographics across the two groups

A significant association was found between TM perforation and the type of procedure performed (p = 0.000) based on the t-test and chi-square test. Specifically, 95.2% (40/42) of patients who underwent tympanoplasty without ossiculoplasty had TM perforation, compared to 43.2% (16/37) of those who underwent tympanoplasty with ossiculoplasty (Table 2).

**Table 2:** Demographics, clinical findings, and scoring systems across the groups TM, tympanic membrane

Variable	Ossiculoplasty (n = 37)	Tympanoplasty w/o ossiculoplasty (n = 42)	p-value	Test statistic
Age (mean ± SD)	40.94 ± 16.34	42.43 ± 17.49	0.473	t(77) = -0.724
TM perforation, n (%)			0	χ²(1) = 22.151
Yes	16 (43.2%)	40 (95.2%)		
No	21 (56.8%)	2 (4.8%)		
Otorrhea, n (%)			0.817	χ²(1) = 0.058
Absent	22 (59.5%)	27 (64.3%)		
Present	15 (40.5%)	15 (35.7%)		
Cholesteatoma, n (%)			0	χ²(1) = 39.811
Yes	20 (54.1%)	1 (2.4%)		
No	17 (45.9%)	41 (97.6%)		
Ossicular chain score, n (%)			0	χ²(3) = 83.353
0	1 (2.8%)	39 (92.9%)		
1	3 (8.3%)	3 (7.1%)		
3	21 (58.3%)	0 (0.0%)		
4	11 (30.6%)	0 (0.0%)		
Missing cases (5)	1 (2.8%)	39 (92.9%)		
Ossicular chain score (mean ± SD)	3.05 ± 0.95	0.07 ± 0.26	0	t(77) = 19.161
Middle ear mucosa, n (%)			0.005	χ²(1) = 7.732
Normal	26 (70.3%)	39 (95.1%)		
Granulation/effusion	11 (29.7%)	2 (4.9%)		
Previous surgery, n (%)			0.474	χ²(1) = 0.568
Yes	14 (37.8%)	12 (28.6%)		
No	23 (62.2%)	30 (71.4%)		
Smoker, n (%)			0.727	χ²(1) = 0.161
Yes	5 (13.5%)	4 (9.5%)		
No	32 (86.5%)	38 (90.5%)		

Similarly, cholesteatoma was significantly associated with the procedure type (p = 0.003). Cholesteatoma was present in 54.1% (20/37) based on the chi-square independent test of patients in the tympanoplasty-only group, compared to only 2.4% (1/42) in the ossiculoplasty group (Table 2).

Middle ear mucosa status was also significantly related to the type of procedure (p = 0.005). Granulation or effusion was observed in 29.7% (11/37) of patients undergoing tympanoplasty with ossiculoplasty, whereas only 4.9% (2/42) by chi-square of patients undergoing tympanoplasty without ossiculoplasty exhibited these conditions (Table 2).

Furthermore, a significant relationship was identified between the ossicular chain score and the type of procedure (p < 0.001). Patients who underwent tympanoplasty with ossiculoplasty had a higher mean ossicular chain score (3.05 ± 0.95) than those who underwent tympanoplasty without ossiculoplasty (0.07 ± 0.26). Specifically, most patients with an ossicular chain score of 3 or 4 had undergone tympanoplasty with ossiculoplasty, whereas those with a score of 0 predominantly underwent tympanoplasty without ossiculoplasty (Table 2).

No significant differences were found between the two groups regarding age (p = 0.473), smoking status (p = 0.727), otorrhea (p = 0.817), or history of previous ear surgeries (p = 0.474) (Table 2).

Outcomes across groups

A significant difference was observed in preoperative and postoperative ABG values between the two groups. Patients who underwent tympanoplasty with ossiculoplasty had higher preoperative ABG (27.47 ± 14.22 dB) and postoperative ABG (15.02 ± 8.58 dB) compared to those who underwent tympanoplasty without ossiculoplasty (20.35 ± 9.62 dB preoperative, 9.48 ± 6.31 dB postoperative) (Table 3). Additionally, total MERI scores were significantly higher in patients who underwent ossiculoplasty compared to those who underwent tympanoplasty without ossiculoplasty (5.56 ± 2.24 vs. 1.78 ± 1.33, p < 0.001) (Table 3).

**Table 3:** Differences in outcomes across groups ABG, air-bone gap; MERI, Middle Ear Risk Index

Measure	Ossiculoplasty (mean ± SD)	Tympanoplasty without ossiculoplasty (mean ± SD)	p-value	Test statistics (t-test)
Preoperative ABG	27.47 ± 14.22	20.35 ± 9.62	0.033	t(77) = 2.188
Postoperative ABG	15.02 ± 8.58	9.48 ± 6.31	0.001	t(77) = 3.748
Total MERI	5.56 ± 2.24	1.78 ± 1.33	0	t(77) = 6.713
Modified MERI	2.59 ± 1.86	1.85 ± 1.11	0.075	t(77) = 1.795

**Figure 1:**
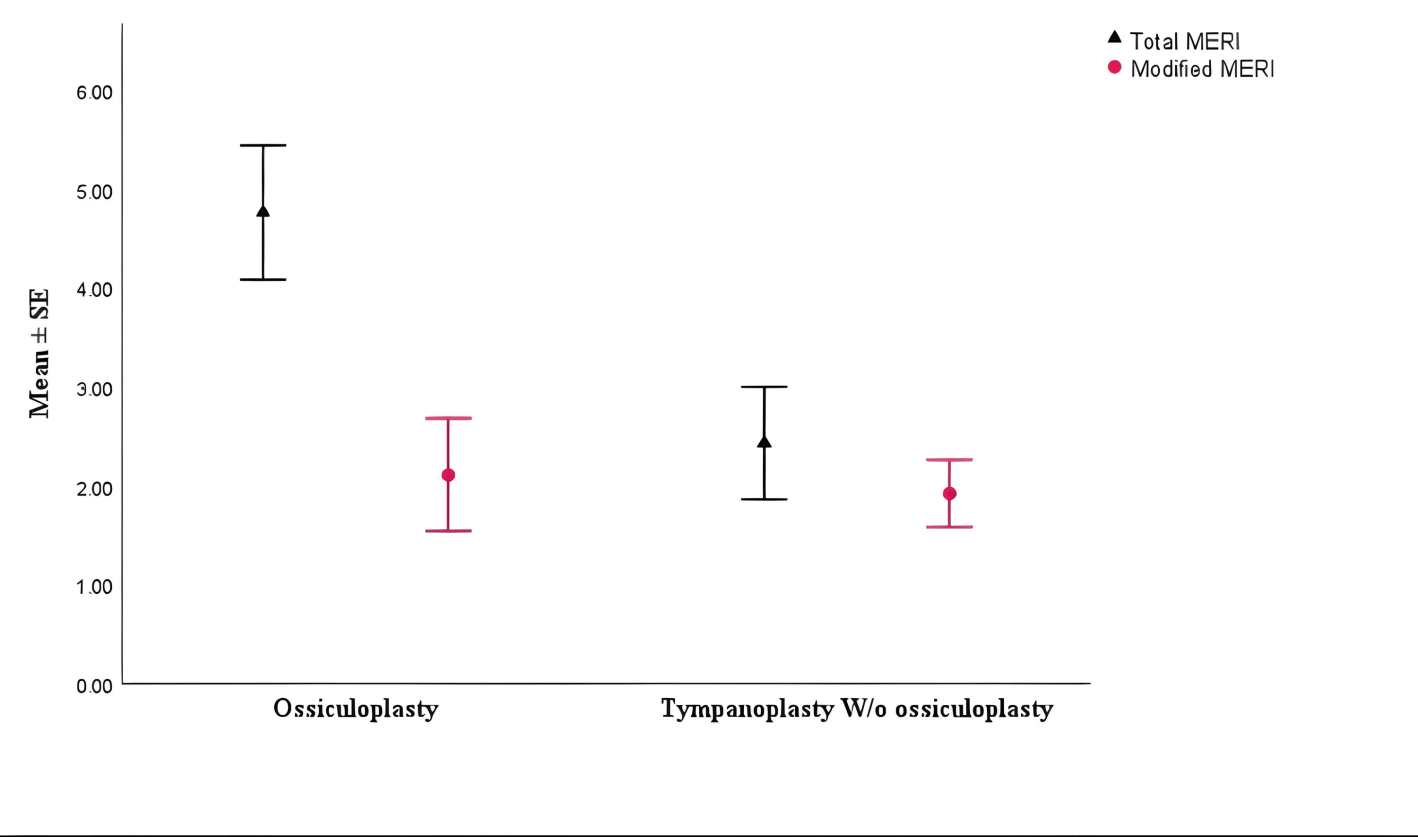
Comparison of total MERI and modified MERI scores between tympanoplasty with ossiculoplasty and tympanoplasty-only groups MERI, Middle Ear Risk Index

A paired sample t-test indicated a significant reduction in ABG post-surgery across both groups, with a mean reduction of 11.76 ± 11.48 dB (p < 0.001). However, no significant differences were found between the modified MERI scores of the two groups (p = 0.844) (Figure 1, Table 3).

Postoperative ABG and ABG differences based on clinical conditions

Analysis of postoperative ABG values revealed that patients with cholesteatoma had significantly higher mean ABG values (16.30 ± 9.88 dB) compared to those without cholesteatoma (10.55 ± 6.50 dB; p = 0.008) (Table 4). Additionally, patients with a history of previous ear surgeries exhibited significantly higher mean postoperative ABG values (15.48 ± 10.00 dB) compared to those without such a history (10.28 ± 5.88 dB; p = 0.013).

**Table 4:** Postoperative ABG and ABG differences are based on clinical conditions ABG, air-bone gap; TM, tympanic membrane

Variable	Postoperative ABG (mean ± SD)	p-value	Test statistics (t-test/ANOVA)
TM perforation		0.091	t(77) = -1.72
Yes	11.15 ± 7.31		
No	14.36 ± 8.98		
Otorrhea		0.437	t(77) = -0.77
Absent	10.85 ± 5.95		
Present	14.05 ± 10.07		
Cholesteatoma		0.008	t(77) = 2.66
Yes	16.30 ± 9.88		
No	10.55 ± 6.50		
Middle ear		0.203	t(77) = -1.29
Normal	11.66 ± 7.86		
Granulation/effusion	14.30 ± 8.46		
Previous surgery		0.013	t(77) = 2.52
Yes	15.48 ± 10.00		
No	10.28 ± 5.88		
Smoker		0.582	t(77) = -0.26
Yes	11.66 ± 4.37		
No	12.14 ± 8.18		
Ossicular chain score		0.002	ANOVA F(3, 75) = 5.21
0	8.81 ± 4.78		
1	13.70 ± 12.00		
3	14.00 ± 5.52		
4	19.00 ± 12.92		

Post hoc analysis demonstrated significant differences in postoperative ABG values between patients with ossicular chain scores of 0 versus 3 (p = 0.007) and 0 versus 4 (p = 0.019) (Table 4).

No significant associations were found between postoperative ABG values and TM perforation (p = 0.091), otorrhea (p = 0.437), middle ear mucosa status (p = 0.203), or smoking status (p = 0.582) all these results are based on paired t-test (Table 4).

Postoperative ABG values and clinical conditions

No significant differences were found in postoperative ABG values between patients with cholesteatoma and those without cholesteatoma, or between patients with and without a history of previous ear surgeries in both the tympanoplasty-only and tympanoplasty with ossiculoplasty groups based on independent t-test (Table 5).

**Table 5:** Postoperative ABG values in both groups across cholesteatoma and previous surgery groups ABG, air-bone gap

Variable	Postoperative ABG in ossiculoplasty (mean ± SD, p-value); test statistics	Postoperative ABG in tympanoplasty without ossiculoplasty (mean ± SD, p-value); test statistics
Cholesteatoma		
Yes	16.76 ± 9.93, 0.367; t(19) = 0.92	23.75 ± 22.98, 0.4; t(24) = 0.84
No	12.96 ± 6.33	9.53 ± 6.38
Previous surgery		
Yes	18.57 ± 10.45, 0.083; t(19) = 1.49	14.03 ± 11.25, 0.094; t(24) = 1.76
No	12.66 ± 6.28	8.42 ± 4.91

Association between cholesteatoma and ossicular chain score

A significant association was identified between the presence of cholesteatoma and ossicular chain score (p < 0.001) with the chi-square test (Table 6). Specifically, patients with cholesteatoma tended to have higher ossicular chain scores, indicating greater ossicular chain involvement.

**Table 6:** Frequency distribution of cholesteatoma across ossicular chain scores

Ossicular chain score	No cholesteatoma (n, %)	Cholesteatoma (n, %)	p-value	Test statistics (chi-square)
0	40 (70.2%)	0 (0.0%)	0	χ²(3, N = 58) = 27.16
1	3 (5.3%)	3 (14.3%)		
3	11 (19.3%)	10 (47.6%)		
4	3 (5.3%)	8 (38.1%)		

Correlation of postoperative ABG values with MERI scores

We created a t-test to assess the significance of the variables. The results are given below.

Both Groups

**Figure 2:**
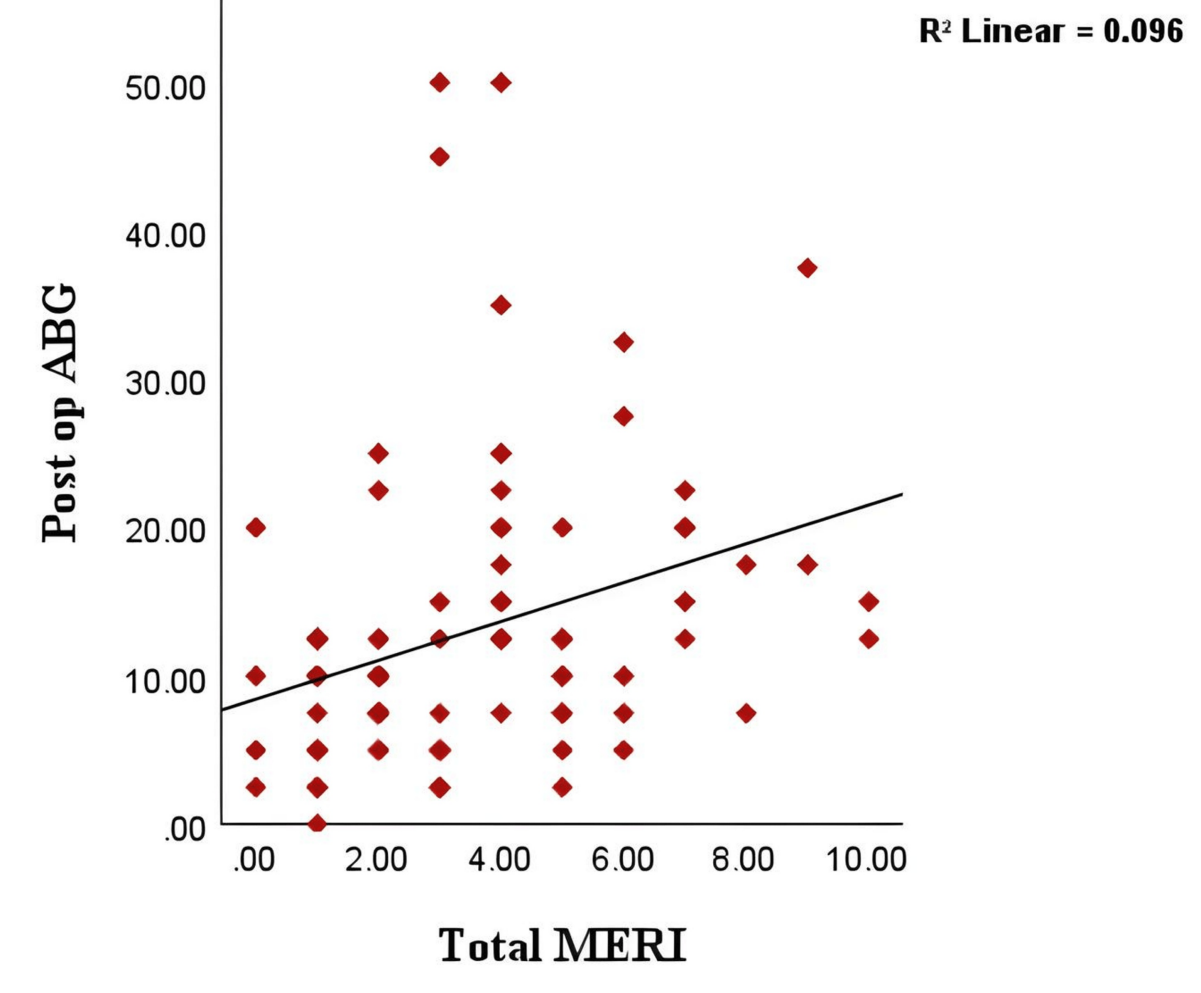
Positive correlation between total MERI scores and postoperative ABG values ABG, air-bone gap; MERI, Middle Ear Risk Index

Bivariate analysis revealed a significant positive correlation between total MERI scores and postoperative ABG values across both patient groups (r = 0.409, p = 0.002) (Table 7, Figure 2). This suggests that higher MERI scores are associated with greater ABG values, indicating poorer hearing outcomes. In contrast, the correlation between modified MERI scores and postoperative ABG values was not statistically significant (r = 0.179, p = 0.126).

**Table 7:** Correlation of postoperative ABG values with MERI scores ABG, air-bone gap; MERI, Middle Ear Risk Index

Variable	Group	Correlation coefficient (r)	p-value	Test statistics	Interpretation
Total MERI	Both groups	0.409	0.002	r(77) = 3.25	Significant positive correlation
Modified MERI	Both groups	0.179	0.126	r(77) = 1.51	No significant correlation
Total MERI	Ossiculoplasty group	0.15	0.105	r(35) = 1.31	No significant correlation
Modified MERI	Ossiculoplasty group	0.12	0.277	r(35) = 1.10	No significant correlation
Total MERI	Tympanoplasty-only group	0.32	0.021	r(41) = 2.38	Significant positive correlation
Modified MERI	Tympanoplasty-only group	0.1	0.871	r(41) = 0.14	No significant correlation
Ossicular chain score	Tympanoplasty-only group	0.454	<0.001	r(41) = 3.90	Significant positive correlation

Ossiculoplasty Group

Within the tympanoplasty with ossiculoplasty group, no significant correlation was found between total MERI scores and postoperative ABG values (r = 0.150, p = 0.105), nor between modified MERI scores and ABG values (r = 0.120, p = 0.277) (Table 7).

Tympanoplasty-Only Group

In the tympanoplasty-only group, a significant association was found between total MERI scores and postoperative ABG values (r = 0.320, p = 0.021), whereas no significant association was observed between modified MERI scores and ABG values (r = 0.100, p = 0.871) (Table 7). Additionally, a significant correlation was identified between ossicular chain scores and postoperative ABG values (r = 0.454, p < 0.001) (Table 7).

## Discussion

Tympanoplasty, with or without ossiculoplasty, is a commonly performed surgical procedure in otolaryngology practice. The main goals of such a procedure are to reduce the recurrence of infections and restore middle ear sound conduction function by reconstructing the ossicular chain [23]. This study included 42 patients who underwent tympanoplasty without ossiculoplasty and 37 who underwent tympanoplasty with ossiculoplasty. We found a significant difference between the two groups in terms of rate of TM perforation, middle ear mucosal disease, cholesteatoma, and ossicular chain score. Most (95.2%) patients who underwent tympanoplasty without ossiculoplasty had TM perforation, while 54.1% of patients who underwent tympanoplasty with ossiculoplasty had cholesteatoma. In addition, those patients also had higher ossicular chain scores.

The MERI score allows surgeons to assess patients based on their preoperative circumstances and anticipate hearing results and risks for complications or recurrence. Kotzias et al. reported a significant correlation between low risk in the MERI scores and ossiculoplasty outcomes [22]. In our study, patients who underwent tympanoplasty with ossiculoplasty had a significantly higher mean MERI score when compared with patients who underwent the procedure without ossiculoplasty (5.56 and 1.78, respectively). We found that the presence of cholesteatoma, advanced ossicular chain score, and history of previous surgery had significantly affected the hearing outcomes, while TM perforation, otorrhea, middle ear granulation of effusion, and smoking had no impact on hearing results. Studies reported various results regarding the factors that affect hearing results. Jung et al. (2021) conducted a retrospective study to assess the effectiveness of the MERI score in predicting the outcomes of 526 patients who underwent tympanoplasty [24]. They found that smoking, otorrhea, and middle ear mucosa status components of the MERI score were associated with an acceptable success rate, whereas performing tympanoplasty with ossiculoplasty was associated with a poor success rate.

Dornhoffer and Gardner reported the prognostic factors of 200 ossiculoplasties and concluded that mucosal status, presence of the malleus handle, otorrhea, mastoidectomy, and revision surgery were significant prognostic factors [25]. Following this, Becvarovski and Kartush proposed a new MERI rating system that includes smoking [26]. Furthermore, further research has confirmed its significance. Pinar et al. enrolled 231 individuals who underwent tympanoplasty [27]. They looked at the MERI score but at other factors as well, and these were found to be significant, such as the size of the perforation, a healthy ear on the other side, and a dry period, which were all linked to the surgical outcome. Furthermore, Castro Sousa et al. found that the quality of the middle ear mucosa has a significant correlation with hearing outcomes [28]. Contrary to these results, Dornhoffer and Gardner found that the middle ear mucosa status had no impact on hearing results [25].

Surgical results are considered successful when postoperative ABG is less than or equal to 20 dB [28]. In our study, patients who underwent tympanoplasty without ossiculoplasty had mean pre- and postoperative ABG values of 20.35 ± 9.62 and 9.48 ± 6.31 dB, respectively, while those who underwent tympanoplasty with ossiculoplasty had mean pre- and postoperative ABG values of 27.47 ± 14.22 and 15.02 ± 8.58 dB, respectively. Success results were achieved in 83.6% and 92.8% in ossiculoplasty and myringoplasty, respectively. Comparing hearing results of ossiculoplasty to simple myringoplasty gives us a more reasonable control group as, even in the presence of an anatomically intact ossicular chain, the success rate of simple myringoplasty is never 100% (around 93% in our series) and the mean ABG is never 0 (around 9.5 dB in our series). Although patients in both groups had a decline in ABG postoperatively, patients who underwent tympanoplasty with ossiculoplasty experienced a significant improvement in ABG and hearing function. This finding is in line with those from previous studies [22,28,29]. Castro Sousa et al. reported the outcomes of 153 ossiculoplasty surgeries [28]. They found that differences in the ABG values did not differ between patients with and without cholesteatoma (p = 0.859). However, normal middle ear mucosa before ossiculoplasty was significantly associated with differences in ABG improvements (p = 0.003). Furthermore, they found a significant post-surgical improvement in ABG after (pre-surgical ABG: 35.3±10.6 dB; post-surgical ABG: 14.0 ± 10.5 dB; p < 0.05). De Corso et al. conducted a retrospective review of 142 patients who underwent tympanoplasty with ossiculoplasty to treat chronic otitis media with cholesteatoma [29]. They found that tympanoplasty with ossiculoplasty was associated with a significant decline in the ABG values (preoperative 28.83 dB; postoperative 13.94 dB). They reported that the percentage of patients with ABG values between 0 and 10 increased from 2.4% to 32.53% after surgery, while those who had ABG values between 11 and 20 dB increased from 16.86% to 37.34%. They concluded a significant improvement in hearing results had occurred after tympanoplasty with ossiculoplasty even when patients had preoperative cholesteatoma. Finally, Kotzias et al. reviewed data from 68 patients who underwent ossiculoplasty between 2006 and 2016 [22]. The mean preoperative ABG was 34.63 dB and decreased to 17.26 dB after surgery, indicating an improvement of 17.36 dB.

We know that interrelated severity factors cannot be simply added up to predict total severity scores. The effect of cholesteatoma + effect of the ossicular chain is not equal to the effect of both factors present at the same time. Our study shows a high level of correlation between the presence of cholesteatoma and a high ossicular chain score (among other factors of the MERI score), and as such, adding up the scores of each of these factors exaggerates the total severity score.

When the OOPS score was described by Dornhoffer, he assumed that the components of his score were not interrelated based on the low level of correlation (but not the zero level of correlation). Interestingly, cholesteatoma was not among the factors he studied in his score. However, other factors might still be interrelated. He tried to establish some degree of linearity between the OOPS score and the postoperative ABG. A careful review of his curve shows that the postoperative ABG was almost the same between scores of 3 and 6. He mentions as well that some patients were excluded from his chart, which further complicates the picture and reduces the reliability of such scores in predicting postoperative ABG.

Our study showed a poor linear correlation between postoperative ABG and the total MERI score, despite being statistically significant (r = 0.4, p = 0.002), a better but still bad correlation between the ossicular chain score and the postoperative ABG (r = 0.45, p = 0.000), and an absence of correlation between the modified MERI score and the postoperative ABG.

In fact, Dornhoffer and Gardner found that stapes were not very significant in affecting hearing results and that only malleus was significant [25], but in a later study he found that even the malleus was not very significant [30]. Our study showed some relation between the total ossicular chain score and hearing result, but a poor relation, as previously mentioned.

We believe that combining independent factors related to poor eustachian tube function might never be a good predictor of postoperative ABG. We believe that studying simple factors might be a better approach as prognostic factors without committing to some theoretical linear relationship.

A key strength of this study is the comprehensive assessment of both demographic and clinical variables, allowing for a nuanced understanding of factors influencing surgical outcomes. The application of uniform metrics, such as MERI and ABG, allows consistency of measurement compared to other works. However, this study also has its limitations, including the relatively small sample size, which may reduce the generalizability of the findings. In addition, being retrospective, there might be selection biases that are difficult to tackle without a large sample size; long-term follow-up data are also not available to comment on the durability of hearing improvements. Therefore, these findings should be verified using larger prospective groups in future studies to enhance validity and generalizability. Longitudinal studies should also be considered to assess the longer-term efficacy of tympanoplasty with ossiculoplasty. The use of the modified MERI scores in other populations may further lead to detail regarding which components most greatly affect hearing outcomes.

## Conclusions

Tympanoplasty with or without ossiculoplasty still represents a challenge in clinical practice; although our understanding of its techniques and influencing factors is improving, the hearing results can be highly successful in cases of ossiculoplasty and are not very far from the control (myringoplasty results). Actual predicting scores are not very successful in predicting outcomes. Simple factors might be more powerful than combination scores. However, larger prospective studies with long follow-ups should be conducted to verify these findings and to better understand the real factors associated with failure. In addition, further modification of MERI scores in larger populations may enhance understanding of the critical factors for optimal results.
